# Epigallocatechin Gallate-Mediated Cell Death Is Triggered by Accumulation of Reactive Oxygen Species Induced via the Cpx Two-Component System in *Escherichia coli*

**DOI:** 10.3389/fmicb.2018.00246

**Published:** 2018-02-15

**Authors:** Tao Nie, Chenlu Zhang, Antian Huang, Ping Li

**Affiliations:** ^1^Research Center for Translational Medicine at Shanghai East Hospital, School of Life Sciences and Technology, Tongji University, Shanghai, China; ^2^School of Life Sciences, Tsinghua University, Beijing, China; ^3^Key Laboratory of Insect Developmental and Evolutionary Biology, Shanghai Institute of Plant Physiology and Ecology, Chinese Academy of Sciences, Shanghai, China

**Keywords:** epigallocatechin gallate, *Escherichia coli*, two-component system, reactive oxygen species, antimicrobial

## Abstract

The high antimicrobial activity of epigallocatechin gallate (EGCG), the most bioactive component of tea polyphenol with a number of health benefits, is well-known. However, little is known about the mechanism involved. Here, we discovered the relationship between reactive oxygen species (ROS), the Cpx system, and EGCG-mediated cell death. We first found an increase in ampicillin resistance as well as the transcription level of a LD-transpeptidase (LD-TPase) involved in cell wall synthesis; *ycbB* transcription was upregulated whereas that of another LD-TPase, *ynhG*, appeared to be constant after a short exposure of *Escherichia coli* to sub-inhibitory doses of EGCG. Additionally, the transcription level of *cpxP*, a downstream gene belonging to the Cpx regulon, was positively correlated with the concentration of EGCG, and significant upregulation was detected when cells were treated with high doses of EGCG. Through analysis of a *cpxR* deletion strain (Δ*cpxR*), we identified a constant ROS level and a notable increase in the survival rate of Δ*cpxR*, while the ROS level increased and the survival rate decreased remarkably in the wild-type strain. Furthermore, thiourea, which is an antioxidant, reduced the ROS level and antimicrobial activity of EGCG. Taken together, these results suggest that EGCG induces ROS formation by activating the Cpx system and mediates cell death.

## Introduction

In *Escherichia coli*, a typical Gram-negative bacterium, there are at least five envelope stress response systems (ESRSs) including Cpx, σE, BaeSR, Psp, and Rcs (Bury-Mone et al., 2009). Among them, the Cpx system, consisting of the histidine kinase CpxA and the response factor CpxR, is a two-component signal transduction system. CpxA, which is located in the inner membrane, senses protein misfolding in the periplasm and autophosphorylates when activated by inducing signals such as pH, osmolarity, or misfolded P pilus subunits. Subsequently, CpxR is phosphorylated by the autophosphorylated CpxA (Raivio and Silhavy, 1997). Phosphorylation enables CpxR to regulate expression of many genes involved in degrading and folding of proteins (*degP, dsbA*, and *ppiA*, etc.) (Danese et al., 1995; Danese and Silhavy, 1997; Pogliano et al., 1997) or cell wall synthesis (*ycbB*) (Bernal-Cabas et al., 2015; Delhaye et al., 2016).

Antimicrobial agents, including antibiotics and some natural products, have been used for many decades in healthcare and cleaning products. Most of their antimicrobial mechanisms have been well-studied, especially for antibiotics (Kohanski et al., 2010). For example, β-lactams can suppress cross-linking of the bacterial cell wall by targeting penicillin-binding proteins (Kohanski et al., 2010). Quinolone-type antibiotics can inhibit DNA replication by targeting DNA topoisomerase IV/DNA gyrase–DNA complexes (Dwyer et al., 2007). Other antibiotics, such as rifamycins and macrolides, inhibit RNA transcription and protein translation, respectively (Kohanski et al., 2010). In addition, it has been clarified that aminoglycosides can bind to the 30S ribosomal subunit and therefore interrupt bacterial protein synthesis, which could further trigger the two-component Cpx system and induce reactive oxygen species (ROS) formation (Kohanski et al., 2008).

Compared with commercial antibiotics, little is known about the antimicrobial mechanism of natural products. For instance, epigallocatechin gallate (EGCG), the most effective antimicrobial constituent in tea polyphenols, has an inhibitory effect on both Gram-positive and Gram-negative bacteria (Steinmann et al., 2013; Reygaert, 2014); however, the antimicrobial mechanism of EGCG is still poorly understood. It has been reported that EGCG causes cell membrane damage and inhibits the activity of several enzymes (Sirk et al., 2008, 2009). EGCG can suppress the activity of bacterial reductases, such as FabG and FabI, leading to inhibition of fatty acid synthesis (Zhang and Rock, 2004). It has also been reported that EGCG is able to inhibit the activity of DNA gyrase by interacting with its ATP binding site (Gradisar et al., 2007). A recent study has shown that EGCG can induce cellular oxidative stress (Xiong et al., 2017b), suggesting that the compound might inhibit bacterial growth through a secondary effect rather than a primary effect. However, the molecular mechanism of EGCG-induced oxidative stress formation is still not very clear.

In this study, we found that sub-inhibitory doses of EGCG could differentially regulate the expression of several cell-wall-related genes that are controlled by the Cpx two-component system. We established that the Cpx system can be activated by EGCG treatment, leading to the accumulation of endogenous ROS and therefore cell death, a novel antimicrobial mechanism for EGCG.

## Materials and Methods

### Materials and Growth Media

Ampicillin was purchased from Sangon, China. Spectinomycin was purchased from ReBio Biotechnology, China. EGCG was purchased from Push Biotechnology, China. 2′,7′-Dichlorofluorescin diacetate (DCFH-DA) was purchased from Sigma, United States. Bacteria were grown in M9 medium (Jensen and Gerdes, 1995) or in LB medium (Xiong et al., 2017b).

### Bacterial Strains

The wild-type *E. coli* strain W3110 (WT, ATCC 27325) was purchased from ReBio Biotechnology, China. Δ*cpxR* is a derivative of the K12 strain W3100 (WT). For generating Δ*cpxR*, a scarless knockout method combining -Red recombination and I-SceI Cleavage was applied (Yang et al., 2014). Two plasmids were used in this study including pREDI and pXX22 (apramycin resistance template plasmid) (Xue et al., 2015). These two plasmids were kindly gifts from Qin Lab (Institute of Plant Physiology and Ecology, Shanghai Institutes for Biological Sciences, Chinese Academy of Sciences). The knockout oligonucleotide primers are listed in **Table 1**.

**Table 1:** Primers sequences for generating Δ*cpxR.*

Gene	Sequence (5′→3′)	Reference or source
Marker	Forward: TAGGCAGCTTAACCGCGCGCAT CTTCGCCATCTTCTGGCTGACGCTGGCG CTGGTGCCTCGAGGTCG	This study
	Reverse: GTTGGGTAACGCCAGGGTTTTC	
Upstream arm	Forward: TGCGGTGACAAGGCGATG	This study
	Reverse: TGGCGAAGATGCGCGCGGTTAA GCTGCCTATACCTCCGAGGCAGAAATTACGTC	
Downstream arm	Forward: GTGACTGGGAAAACCCTGGCGT TACCCAACACTTGATCGCGTTCTCGG	This study
	Reverse: CACGGGTGACCATCTTTAC	

### Bacterial Culture and EGCG-Adaption Conditions

For *E. coli* cultivation, bacteria were diluted to 2% in M9 medium and shaken at 180 rpm until the cells reached mid-logarithmic phase (OD_600_ of 0.5). For EGCG adaption, bacteria were cultured in M9 medium to an OD_600_ of 0.1 (about 10^7^ CFU/ml) before the addition of 400 μg/ml EGCG (Serra et al., 2016). The samples were continuously cultured to reach an OD_600_ of 0.5 (about 2 h). All concentrations of EGCG quoted were final concentrations. All bacterial cultures and incubations were conducted at 37°C.

### Antimicrobial Assays

For antibiotic susceptibility tests, EGCG-adapted bacteria were washed twice with saline (0.8% NaCl) then uniformly plated on LB agar; the disk diffusion test was then performed on the basis of Clinical and Laboratory Standards Institute guidelines (CLSI, 2011). For determining survival rates, bacteria were cultured to an OD_600_ of 0.5, then EGCG at various concentrations (from 1 to 2 mg/ml) with or without thiourea (125 mM) (Dorsey-Oresto et al., 2013) or catalase (20 μg/ml) (Kolodkin-Gal et al., 2008) was added to the broth and the bacteria were cultured for a further 6 h. The bacteria were then washed twice with saline and plated on LB agar after diluting by 10^5^-fold. The LB agar plates were cultured at 37°C overnight, and the number of colonies formed was counted.

### RNA Isolation

Bacteria were cultured to an OD_600_ of 0.5. Different amounts of EGCG (1.2 and 1.6 mg/ml) were added and incubated with the bacteria for 6 h. These bacteria and the EGCG-adapted bacteria were washed twice with saline. Total RNA was isolated based on the protocol of RNAiso Plus 9180 (TaKaRa, China). RNA concentrations were measured with a Nanodrop (Thermo, United States), and 500 ng of total RNA was reverse-transcribed using a FastQuant RT kit (TianGen, China).

### Reverse-Transcription (RT)-PCR

The quantitative PCR (qPCR) reaction mixture contained 10 μl SGExcel FastSYBR Mixture (Sangon, China), 0.5 μl of each primer (10 μM), 0.5 μl template cDNA, and double-distilled H_2_O up to 20 μl. The transcription level of the 16S rRNA gene was used to normalize the transcription levels of target genes. Primers used are listed in **Table 2**. qPCR amplification was carried out on an ABI 7500 detection system (Applied Biosystems, United States) using a three-step method with an annealing temperature of 56°C and 40 amplification cycles in total.

**Table 2:** Primer sequences for qPCR.

Gene	Sequence (5′→3′)	Reference or source
*ycbB*	Forward: GCAGAAACTAAGCCGATGGA	This study
	Reverse: ACGGCTTCCACCAGTTCAT	
*ynhG*	Forward: CGGACAGCGTCGGTTTG	This study
	Reverse: CGTTAGGCTCCACGGAAT	
*cpxP*	Forward: TAAGCCACGCTGCTGAAGT	This study
	Reverse: TTTGCTCATTCGCCATTTT	

### Measurement of ROS

Bacterial cells with an OD_600_ of 0.5 were treated with 1.6 mg/ml EGCG. ROS level was measured from 1 to 6 h. After being washed twice with saline, the bacteria were incubated with DCFH-DA at a final concentration of 20 μM for 30 min in the dark. Subsequently, the bacteria were washed twice with saline and subjected to analysis using a SpectraMax M5 (Molecular Devices, United States) for determining the ROS level at 488 nm excitation and 525 nm detection.

### Statistical Analysis

At least three biological replicates were performed for each experiment. Data are given as means ± standard deviation (SD). All comparisons to determine differences were performed by applying Student’s *t*-test and one-way analysis of variance. A *P*-value < 0.05 was considered significant.

## Results

### A Sub-inhibitory Dose of EGCG Increases the Resistance of *E. coli* to β-Lactam Ampicillin

**FIGURE 1:**
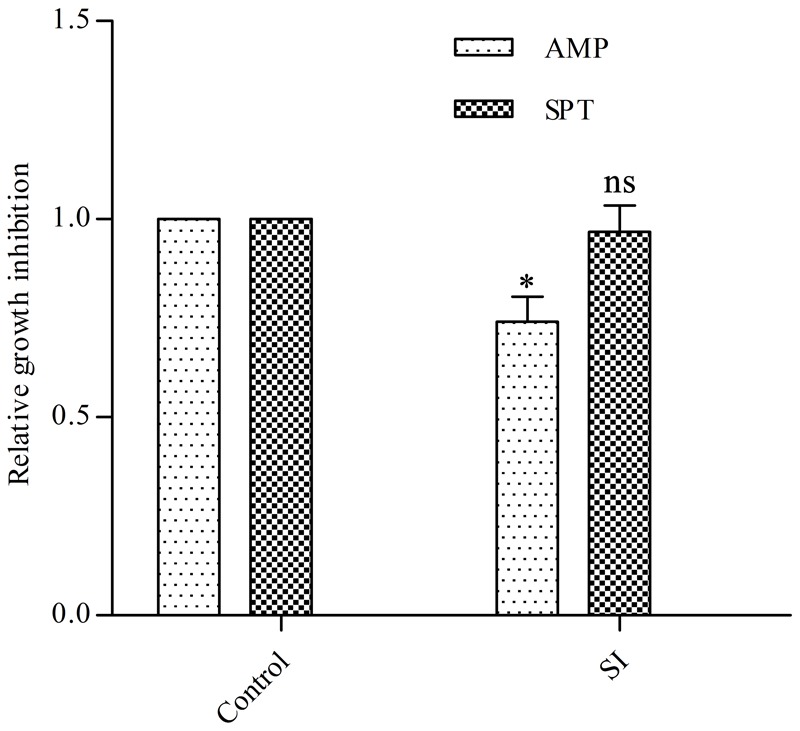
Effects of EGCG on antibiotic resistance and cell survival. A sub-inhibitory (SI) dose of EGCG (400 μg/ml) increases resistance to ampicillin. The growth inhibition diameter was measured around disks containing 20 μg ampicillin (AMP) or 100 μg spectinomycin (SPT). Cells without EGCG treatment were used as a control. *y* axis represents values normalized by the average diameter obtained for the control. All values were obtained from at least three biological replicates. Error bars indicate standard deviation (SD). ^∗^*P* < 0.05. ns, not statistically significant.

Firstly, we determined the minimum inhibitory concentration (MIC) of EGCG on *E. coli* (1.2 mg/ml). To test the effect of EGCG on the resistance of *E. coli* to antibiotics we used the disk diffusion test to examine the antibiotic susceptibility of cells treated with sub-inhibitory doses of EGCG (400 μg/ml). As expected, cells showed increased resistance to ampicillin but not to spectinomycin (**Figure 1**). These results indicated that EGCG might have an effect on the bacterial cell wall.

### EGCG Influences the Transcription of Cell-Wall-Related Genes

**FIGURE 2:**
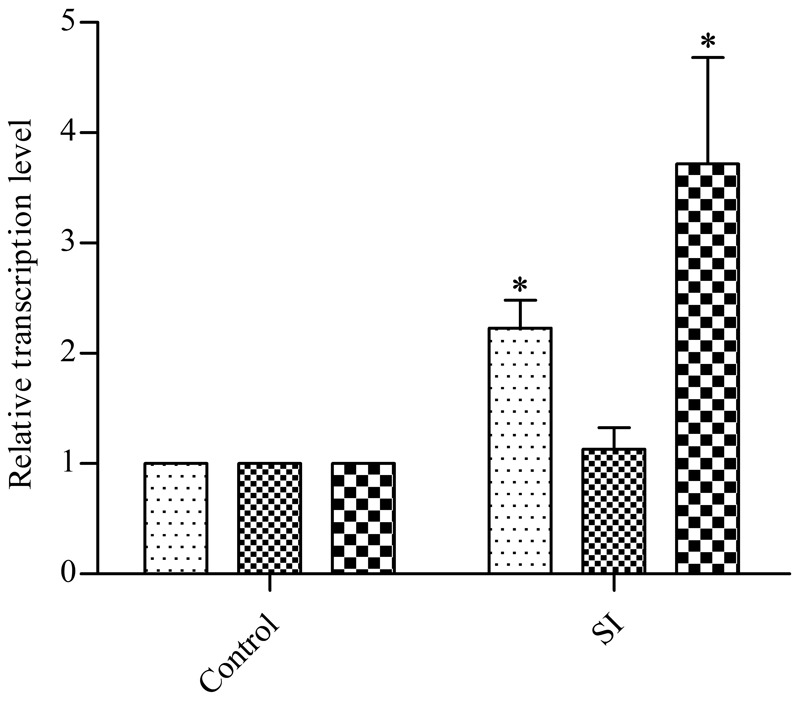
Effects of a sub-inhibitory dose of EGCG on the transcription levels of cell-wall-related genes. As a control, cells without EGCG treatment were cultured until OD_600_ reached 0.5. *y* axis represents comparative critical threshold values obtained after normalizing data against 16S rRNA gene. All values were obtained from at least three biological replicates. Error bars indicate SD. ^∗^*P* < 0.05.

Due to the increased resistance of *E. coli* to β-lactam ampicillin, we determined the transcription levels of cell-wall-related genes. Previous studies have shown that the LD- transpeptidases (LD-TPase) YcbB and YnhG are involved in forming β-lactam-insensitive peptide crosslinks (Typas et al., 2011), so we tested these two genes by using qRT-PCR. After treatment with 400 μg/ml EGCG, *ycbB* was upregulated (ca. twofold) while the transcription level of *ynhG* remained unchanged (**Figure 2**), which may indicate YcbB is relatively more important than YnhG when exposed to EGCG. *ycbB* is reported to be a downstream gene of the Cpx two-component system (Bernal-Cabas et al., 2015; Delhaye et al., 2016); hence, we also examined the transcription level of *cpxP*, which is positively regulated by the Cpx system (Danese and Silhavy, 1998), and found a fivefold upregulation of *cpxP* when compared with the mock control (**Figure 2**). These data indicated that sub-inhibitory doses of EGCG could activate the Cpx system in *E. coli*.

### EGCG Triggers Cell Death by Activating the Cpx System

**FIGURE 3:**
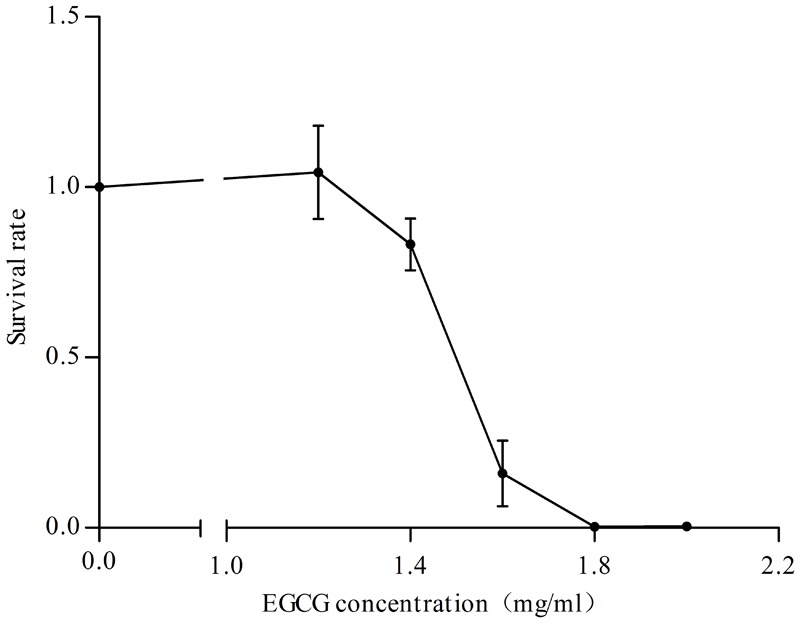
Effects of various concentrations of EGCG on cell survival. Cells without EGCG treatment were used as a control. *y* axis represents the relative survival rate normalized by the average value of total cells obtained for the control. All values were obtained from at least three biological replicates. Error bars indicate SD.

**FIGURE 4:**
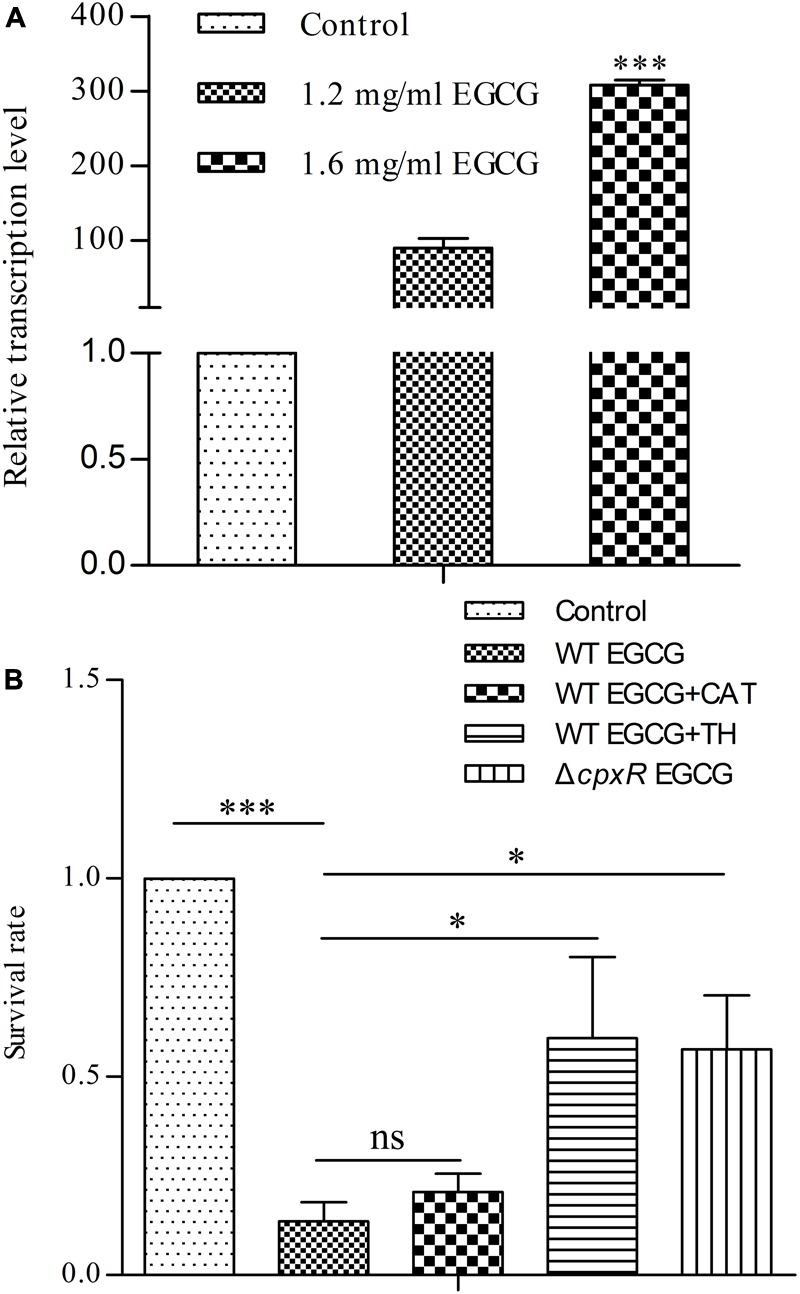
Cpx system activated by EGCG induces cell death. **(A)** The Cpx system is activated by EGCG. As a control, cells with an OD_600_ of 0.5 were cultured for 6 h. *y* axis was described in **Figure 2**. **(B)** Effects of reactive oxygen species (ROS) on EGCG-mediated cell death. WT EGCG, WT EGCG+CAT, and WT EGCG+TH represent WT exposed to 1.6 mg/ml EGCG, 1.6 mg/ml EGCG plus 20μg/ml catalase and 1.6 mg/ml EGCG plus 125 mM thiourea, respectively. Δ*cpxR* EGCG represents Δ*cpxR* exposed to 1.6 mg/ml EGCG. Cells without EGCG treatment were used as a control. *y* axis was described in **Figure 3**. All values were obtained from at least three biological replicates. Error bars indicate SD. ^∗^*P* < 0.05; ^∗∗∗^*P* < 0.001. ns, not statistically significant.

Since the Cpx system acts as a critical regulator in maintaining envelope integrity (Gerken et al., 2010), we also asked whether the Cpx system was activated in response to different concentrations of EGCG. Firstly, we determined the survival rate of cells treated with EGCG. When the concentration of EGCG increased to 1.6 mg/ml, the survival rate was approximately 15% (**Figure 3**), implying that EGCG has a bactericidal property, similar to results of a previous study (Xiong et al., 2017b). We then analyzed the transcription levels of *cpxP* under 1.2 mg/ml (MIC) and 1.6 mg/ml EGCG. The *cpxP* gene was upregulated in a dose-dependent manner (**Figure 4A**). Strikingly, relative to the mock control, a ca. 300-fold increase in *cpxP* expression was detected in *E. coli* treated with 1.6 mg/ml EGCG (**Figure 4A**). Together with the bactericidal effect of EGCG, we hypothesized that the Cpx system was involved in cell death caused by EGCG. To confirm this theory, a *cpxR* deletion strain (Δ*cpxR*) was constructed and the survival rate of this deletion mutant treated with 1.6 mg/ml EGCG was determined. Surprisingly, a remarkably increased survival rate was observed in Δ*cpxR* treated with EGCG when compared with the WT treated with EGCG (**Figure 4B**), which indicated that the Cpx system contributed to cell death triggered by EGCG.

### Activation of the Cpx System Induces ROS Formation

**FIGURE 5:**
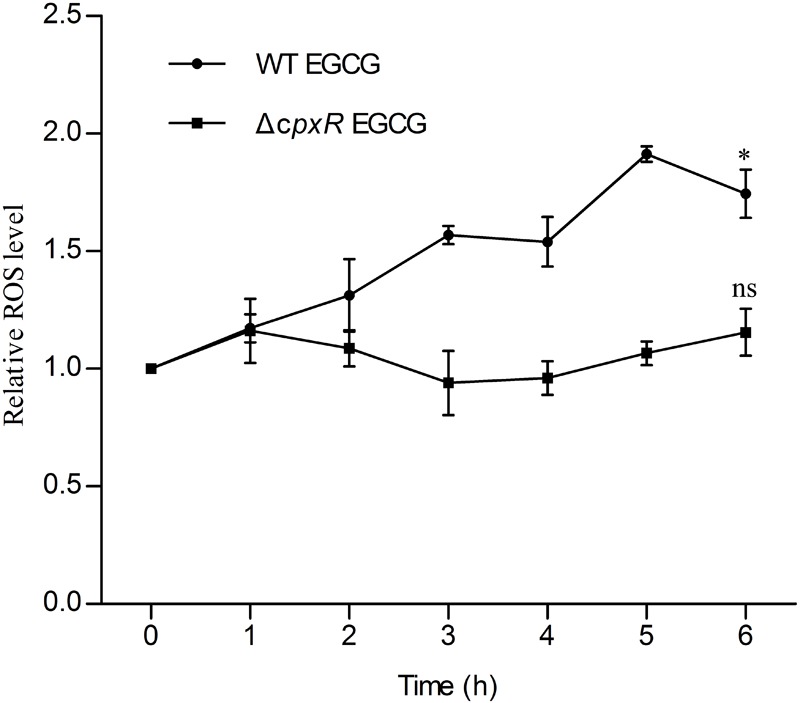
Reactive oxygen species accumulation in WT and Δ*cpxR* upon EGCG treatment. WT EGCG and *ΔcpxR* EGCG represent WT and *ΔcpxR* exposed to 1.6 mg/ml EGCG, respectively. ROS level was measured from 1 to 6 h. Cells, WT or *ΔcpxR*, without EGCG treatment were used as controls. *y* axis represents the relative ROS level normalized by the average value of ROS level obtained for the control. All values were obtained from at least three biological replicates. Error bars indicate SD. ^∗^*P* < 0.05. ns, not statistically significant.

On the basis of all of the above results, we further investigated the relationship between the Cpx system and cell death. Generally, the Cpx system is considered as a way to control protein homeostasis in the cell envelope (Gerken et al., 2010), but it also triggers aminoglycoside-antibiotic-mediated cell death by inducing hydroxyl radical formation (Kohanski et al., 2008). Moreover, a recent study showed that EGCG inhibited a sub-strain of *E. coli*, OP50, by increasing endogenous ROS (Xiong et al., 2017b). Therefore, we suspected that EGCG might act in a similar manner to aminoglycoside antibiotics. Thus, we first detected whether EGCG-mediated cell death correlated with endogenous ROS in WT cells. When exposed to 1.6 mg/ml EGCG together with thiourea, a reductant scavenging endogenous ROS (Dorsey-Oresto et al., 2013), the survival rate of the bacterial cells was significantly increased while it remained unchanged when exposed to EGCG plus catalase (**Figure 4B**) in comparison with the significant decrease in survival observed after treatment with EGCG alone (**Figure 3**). More importantly, ROS levels in both WT and Δ*cpxR* were also examined. Upon exposure to 1.6 mg/ml EGCG, the ROS level in Δ*cpxR* cells remained unchanged but there was a notable increase in a time-dependent manner in the ROS level of WT cells (**Figure 5**). Collectively, these data indicated that endogenous ROS are induced by the activated Cpx system.

## Discussion

Several studies have reported the health benefits of EGCG including antioxidant, anticancer, and antimicrobial properties (Yang et al., 2009; Kim et al., 2014; Reygaert, 2014). Recently, it was also found that EGCG could promote a healthy lifespan in *Caenorhabditis elegans* (Xiong et al., 2017a). In this study, we found a novel bactericidal mechanism of EGCG in which EGCG triggers cell death via induction of ROS formation by activation of the Cpx system.

Previous evidence has indicated that many stresses can increase the resistance of bacteria to antibiotics (Poole, 2012). For example, antibiotics at sub-lethal levels stimulate bacterial resistance (Harms et al., 2016). Indeed, in our work, we found that 400 μg/ml EGCG elevated the resistance of *E. coli* to β-lactam, but not to spetinomycin or norfloxacin (data not shown), similar to a study demonstrating that EGCG at a sub-inhibitory dose enhances resistance to β-lactam by activating the VasRA system in *Staphylococcus aureus* (Levinger et al., 2012). Moreover, we observed that *ycbB* was upregulated. Since YcbB is considered to be a LD-TPase, which is involved in forming the minor type of β-lactam-insensitive peptide crosslinks (Typas et al., 2011), upregulation of *ycbB* might result in increased resistance to β-lactams. Besides, EGCG shows specific binding to peptidoglycan that may contribute to this result as well (Zhao et al., 2001). *ycbB* is a downstream gene in the Cpx regulon in *E. coli* (Bernal-Cabas et al., 2015; Delhaye et al., 2016). When *E. coli* was exposed to EGCG, the Cpx system was activated in a dose-dependent manner. Generally, the Cpx system and the σE pathway are viewed as regulators responding to disturbances including misfolded proteins in the cell envelope, and these two regulons share some activators and target genes (Ruiz and Silhavy, 2005). Our results are consistent with a recent study demonstrating that EGCG activates the σE pathway, blocking the transcription of *csgD* which encodes a biofilm regulator and inhibiting the formation of biofilm in *E. coli* since *csgD* is negatively regulated by the Cpx system and σE pathway (De Wulf et al., 2002; Serra et al., 2016). Moreover, EGCG elevated the resistance to ampicillin but not to spectinomycin. Together with several studies showing that activating the Cpx system increases the resistance to some but not all antibotics (Mahoney and Silhavy, 2013; Delhaye et al., 2016), we propose that EGCG increases the resistance to ampicillin through the Cpx system.

Previously, many studies have focused on the antimicrobial activity of EGCG including damage to the cell membrane, and inhibition of fatty acid synthesis and the activity several enzymes (Zhang and Rock, 2004; Sirk et al., 2008, 2009). A recent report found the inhibitory effect of EGCG might be a result of endogenous ROS and that EGCG could trigger an antioxidant response as well as induce ROS formation in a time-dependent manner (Xiong et al., 2017b). In our study, a similar result was obtained that the bactericidal effect of EGCG was significantly decreased when EGCG was used together with thiourea instead of catalase. Moreover, a notable accumulation of ROS was observed in our study. These findings together with our results imply that endogenous ROS plays a key role in EGCG-mediated cell death. As regards the mechanism of ROS formation, many studies have been carried out on antibiotics. A typical mechanism of cell death mediated by ROS is the MazF-Cpx-ROS pathway (Zhao and Drlica, 2014). MazEF is a toxin-antitoxin system in *E. coli*. Within this system, MazF is an endonuclease (toxin) cleaving single-stranded mRNAs at ACA nucleotide sequences while MazE is a labile antitoxin, which can be degraded by the protease ClpAP. Normally, MazE binds to MazF inhibiting its endonuclease activity (Ramisetty et al., 2015). When *E. coli* is exposed to antibiotics that inhibit transcription or translation, MazE is degraded faster than it is synthesized and thereby MazF toxin is liberated. The liberation causes MazF to cleave mRNA, which leads to production of mistranslated (misfolded) proteins. Subsequently, the Cpx system is activated through detection of misfolded proteins in the cell membrane. The Cpx system then induces ROS formation by coupling with the ArcAB system through the tricarboxylic acid (TCA) cycle (Zhao and Drlica, 2014). However, other antibiotics including β-lactams and quinolones also trigger ROS formation through the Cpx and ArcAB systems although they cannot liberate MazF (Kohanski et al., 2008). Hence, the Cpx system may be an important element in ROS formation. Indeed, EGCG decreases the expression of proteins involved in the TCA cycle (Yi et al., 2010), and it seems that EGCG acted in a similar manner to β-lactams or quinolones in our study since it activated the Cpx system inducing ROS formation and triggering cell death in a way that did not involve MazF (data not shown). Furthermore, on the basis of several studies that have showed EGCG interacts with peptidoglycan (Zhao et al., 2001) and outer membrane proteins (Nakayama et al., 2013), we propose that EGCG may induce a disturbance in the cell envelope that activates the Cpx system.

## Conclusion

Our study provides new insights into the action of EGCG, showing that EGCG triggers cell death by inducing endogenous ROS via the Cpx system. Meanwhile, the mechanism of how the Cpx system is activated by EGCG deserves further investigation in the future.

## Author Contributions

TN and PL conceived and designed the experiments. TN and AH performed the experiments. TN analyzed the data. TN, CZ, and PL contributed to the writing of the manuscript.

## Conflict of Interest Statement

The authors declare that the research was conducted in the absence of any commercial or financial relationships that could be construed as a potential conflict of interest.
